# Increases in Stressors Prior to-Versus During the COVID-19 Pandemic in the United States Are Associated With Depression Among Middle-Aged Mothers

**DOI:** 10.3389/fpsyg.2021.706120

**Published:** 2021-07-07

**Authors:** Brittany K. Taylor, Michaela R. Frenzel, Hallie J. Johnson, Madelyn P. Willett, Stuart F. White, Amy S. Badura-Brack, Tony W. Wilson

**Affiliations:** ^1^Institute for Human Neuroscience, Boys Town National Research Hospital, Omaha, NE, United States; ^2^Department of Psychological Science, Creighton University, Omaha, NE, United States

**Keywords:** coronavirus – COVID-19, maternal, stress, anxiety, depression

## Abstract

Working parents in are struggling to balance the demands of their occupation with those of childcare and homeschooling during the COVID-19 pandemic. Moreover, studies show that women are shouldering more of the burden and reporting greater levels of psychological distress, anxiety, and depression relative to men. However, research has yet to show that increases in psychological symptoms are linked to changes in stress during the pandemic. Herein, we conduct a small-*N* study to explore the associations between stress and psychological symptoms during the pandemic among mothers using structural equation modeling, namely latent change score models. Thirty-three mothers completed questionnaires reporting current anxious and depressive symptoms (Beck Anxiety and Depression Index, respectively), as well as stressful life experiences prior to-versus during the pandemic (Social Readjustment Rating Scale). Women endorsed significantly more stressful events during the pandemic, relative to the pre-pandemic period. Additionally, 58% of mothers scored as moderate-to-high risk for developing a stress-related physical illness in the near future because of their pandemic-level stress. Depressive symptoms were associated with the degree of change in life stress, whereas anxiety symptoms were more related to pre-pandemic levels of stress. The present study preliminarily sheds light on the nuanced antecedents to mothers’ experiences of anxious and depressive symptoms during the COVID-19 pandemic. Although further work is needed in larger, more diverse samples of mothers, this study highlights the potential need for appropriate policies, and prevention and intervention programs to ameliorate the effects of pandemics on mothers’ mental health.

## Introduction

The coronavirus disease of 2019 (COVID-19) pandemic has disrupted almost every facet of life across the globe. At the time of this writing, over 100 million people around the world have contracted COVID-19. More than 2.1 million people have died globally, with roughly one-quarter of infections and fatalities occurring in the United States according to the Johns Hopkins Resource Center.^1^ The gravity of the situation has left families in turmoil, and has introduced multiple compounded stressors including social isolation, disruptions to personal routines, revisions to personal hygiene practices, loss of childcare and schooling for youth, and financial and employment uncertainty (Taylor, 2019; Coyne et al., 2020; Fisher et al., 2020; Islam et al., 2020). The effects have been notably compounded in the United States, where dissemination of important health information from leading politicians and news outlets has been mixed and often contradictory to World Health Organization guidelines (Park et al., 2020; Rauch et al., 2020). These stressors have resulted in unprecedented increases in psychological distress (Fitzpatrick et al., 2020; Fullana et al., 2020; Pfefferbaum and North, 2020; Rajkumar, 2020; Salari et al., 2020; Torales et al., 2020; Twenge and Joiner, 2020; Xiong et al., 2020).

In particular, the effect of pandemic-related stressors on parents’ mental health is remarkably high, with multiple studies showing high anxiety and depression among parents of youth in the United States (Brown et al., 2020; Coyne et al., 2020; Drouin et al., 2020; Park et al., 2020; Venkatesh and Edirappuli, 2020). In fact, one study reported that by early June 2020, 27% of parents felt their mental health was generally suffering in light of the pandemic (Patrick et al., 2020). Parents are facing the reality of an unstable economy, which lends itself to job insecurity, financial distress, potential loss of health insurance, and worries over food security (Taylor, 2019; Patrick et al., 2020). Moreover, research shows that working mothers have been disproportionately affected in the current pandemic due to high unemployment in service-based industries, which are largely occupied by women, and due to gender inequality in housework and childcare obligations (Alon et al., 2020; Blundell et al., 2020; Kalenkoski and Pabilonia, 2020; Zamarro, 2020). Stay-at-home orders, and school and daycare facility closures across the country have left families scrambling to manage childcare, and many working mothers report that they are solely or primarily bearing the weight of childcare *in addition to* their own employment obligations (Ma et al., 2020; Patrick et al., 2020; Power, 2020). This challenging work-home balance has resulted in decreased work-related productivity among women (Kibbe, 2020), and increased parental distress (Duan et al., 2020; Spinelli et al., 2020).

In addition to, or perhaps because of these factors, women’s mental health seems to have been more adversely impacted by COVID-19 than men’s mental health (Fitzpatrick et al., 2020; Ho et al., 2020; Hyland et al., 2020; Mazza et al., 2020; Rossi et al., 2020). In fact, the prevalence and severity of pandemic-specific life stressors and psychological symptoms among women is alarming. Prior research has repeatedly demonstrated that even outside of pandemic conditions, increased stress and mental illness are associated with increased risk of contracting *physical* illnesses, including conditions like coronary heart disease, respiratory infections, and autoimmune diseases (Christie-Seely, 1983; Cohen et al., 2019; Cullen et al., 2020). Such stress-induced vulnerability to physical illness during a global pandemic is a public health crisis that requires attention. Interestingly, most studies to date have not assessed *changes* in the amount or impact of life stress prior to-versus during the pandemic. Rather, it is more common to see studies reporting only perceptions and exposures to stress during the pandemic, with little questioning about the degree of change in stressful experiences.

### The Current Study

Herein, we examine the degree to which life stress has changed among parents since the pandemic began, and to what extent that change is associated with psychological consequences, specifically self-reported anxiety and depression symptoms. The present study focuses on reports acquired from mothers between mid-May and late-June 2020. Given the prior literature, we hypothesized that there would be a significant increase in the number of major life events/stressors endorsed among mothers, and therefore a greater impact of life stress. Moreover, we expected that a greater degree of change in life stress would be associated with greater anxious and depressive symptoms reported during the pandemic. We also comment on the potential risk for contracting a physical illness in the near future given the impact of life stressors reported by mothers.

## Materials and Methods

### Participants

Families who were enrolled in ongoing longitudinal studies of neurocognitive development in youth (Taylor et al., 2020; Stephen et al., 2021) were invited to remotely complete online surveys following COVID-19 lockdowns. A total of 37 unique families in the greater Omaha, Nebraska area completed the present sub-study examining social and psychological repercussions of the COVID-19 pandemic. Of these families, 35 respondents were mothers and two were fathers. Due to the limited number of male respondents, and the known sexual disparities in the presentation and experience of pandemic-related stress, we focfused only on data gathered from mothers. Two women did not complete all questionnaires. Thus, the final sample for this study was comprised of 33 middle-aged (*M*_age_ = 45.58 years, SD = 4.64, range = 36–56 years-old) mothers of children and adolescents (*M*_children_ = 2.38 children currently living in the home, SD = 1.10, range = 1–6 children). Thirty-two mothers identified a biological parent of youth, and one identified as an adoptive mother. All women were Caucasian; 32 identified as Non-Hispanic/Latino and one identified as Hispanic/Latino. Twenty-eight mothers provided their highest level of education (*n* = 16 graduate/professional degree; *n* = 10 college; *n* = 2 partial college). Participants electronically signed informed consent forms following a complete description of the protocol. Study procedures were approved by the local university’s Institutional Review Board. The authors assert that all procedures contributing to this work comply with the ethical standards of the relevant national and institutional committees on human experimentation and with the Helsinki Declaration of 1975, as revised in 2008.

### Questionnaires

Parents completed a battery of questionnaires probing repercussions of the COVID-19 pandemic. All questionnaires were delivered electronically *via* the Collaborative Informatics and Neuroimaging Suite (COINS; Scott et al., 2011; Landis et al., 2016) and participants could complete the surveys at their leisure within a one-week span. Some questionnaires were custom-designed and retrospectively probed changes in employment, income, family activities, and childcare responsibilities as a result of the pandemic. Researchers who wish to adopt these measures can request materials from the corresponding author.

In addition to newly-developed instruments, participants completed a modified version of the Social Readjustment Rating Scale (SRRS; Holmes and Rahe, 1967) The traditional SRRS asks respondents to indicate whether each of 43 unique stressful events occurred within the past 12 months. For the present study, we adapted the measure to probe differences in the number of stressful events endorsed prior to versus during/after local lockdowns began. The instructions for the survey read as follows:

“For each event, indicate whether this happened to you in the 6 months prior to when people started staying home (about March 15, 2020), and since people started staying home (since about March 15, 2020).”

For each item in the survey, respondents marked “yes” or “no” to indicate whether they had experienced that stressor in the 6 months prior to March 15, 2020, and/or since March 15, 2020. Thus, all reports of life events prior to the pandemic were retrospective. Our modified survey excluded one item, which asked whether Christmas was approaching, because the study time frame inherently biased responses to this item. From the SRRS, we calculated the total number of stressful events endorsed pre-and post-lockdown. We also computed life stress impact scores using the norms outlined and used in previous studies (e.g., Holmes and Rahe, 1967; Blasco-Fontecilla et al., 2012; Mirabzadeh et al., 2013). Briefly, each stressful event has an associated “stress impact score,” which implies the relative expected effect of that event on a person’s overall health. An individual’s total impact score (the sum of all impact scores for endorsed stressors) has been linked to the overall risk of developing a physical illness in the near future, including such ailments as respiratory infections and autoimmune and coronary diseases (Cohen et al., 1998, 2007, 2019). Scores < 150 are considered “low risk,” with only a 30% change of contracting physical illness in the near future. Stress impact scores between 150 and 299 are associated with a moderate (50%) risk of illness, and scores >300 are associated with an 80% increased risk of experiencing physical illness in the near future (Christie-Seely, 1983; Blasco-Fontecilla et al., 2012; Weber et al., 2013; Figueroa and Zoccola, 2015; Habersaat et al., 2015).

In addition to the SRRS, parents completed the standard, unmodified Beck Depression Inventory (BDI-II; Beck et al., 1996) and Beck Anxiety Inventory (BAI; Beck et al., 1988), which probe symptoms experienced within the past two weeks. The BDI asks respondents to rate on a 4-point scale the extent to which they endorse 21 different feelings, patterns of thought, and behaviors. Similarly, the BAI asks respondents to identify the extent to which they have been bothered by 21 unique physical and psychological manifestations of anxiety, again on a 4-point scale. For both the BAI and BDI, higher total scores are indicative of greater anxious or depressive symptoms, respectively.

### Statistical Analyses

We computed a Wilcoxon Z test to determine whether the number of stressful events differed pre-versus post-lockdown. We hypothesized that participants would endorse a greater number of stressful events post-lockdown relative to pre-lockdown. Next we calculated stress impact scores for the pre-and post-lockdown periods per person based on endorsed stressors. We comment on relative risk for contracting a stress-related physical illness in the near future based on commonly accepted ranges of impact scores (Rahe et al., 1970; Blasco-Fontecilla et al., 2012; Figueroa and Zoccola, 2015).

We then determined the extent to which changes in stress impact scores were associated with self-reported anxiety and depressive symptoms during post-lockdown periods using the BDI and BAI total scores. We expected that individuals with greater increases in stress impact scores would report greater post-lockdown anxiety and depressive symptoms. For a robust analysis of differences in stress impact, we employed a latent change score modeling scheme. We first estimated a model of the latent change in stress impact scores without any other variables in the model using standard parameters (Kievit et al., 2018). Namely, pre-lockdown scores predicted post-lockdown scores, and a latent variable was defined by the post-lockdown score constrained to 1. The mean and variance of the post-lockdown scores were constrained to 0. Pre-lockdown scores were then modeled as a predictor of the latent change variable. This approach allowed us to simultaneously assess: (1) whether there was reliable change in stress impact scores from pre-to post-lockdown periods across the sample, (2) the degree to which changes in stress impact varied across individuals, and (3) the extent to which changes in impact scores were dependent on pre-lockdown stress impact. We then modeled the latent change score as a predictor of BAI and BDI total scores to examine whether changes in life stress were associated with self-reported anxiety and depressive symptoms. BAI and BDI scores were allowed to freely correlate with each other, and with stress impact scores pre-lockdown. Statistical significance was determined based on a threshold of *α* = 0.05. Analyses were completed using MPlus version 8.1.

## Results

### COVID-19 Restrictions in the Area During the Study

As mentioned in the Methods section, all mothers who completed the present study were from the greater Omaha, Nebraska area. A complete timeline of local events, restrictions, and policy changes is documented here.^2^ From late January to early February 2020, 57 patients who tested positive for COVID-19 arrived in Omaha, Nebraska seeking treatment at the University of Nebraska Medical Center Biocontainment Unit. The state of Nebraska had its first public case of COVID-19 on March 6th. Public schools in the greater Omaha area closed their doors mid-March with no plans to return to in-person learning for the foreseeable future, and the governor set restrictions on public gatherings. Graduations, sporting events, and other major gatherings were canceled shortly thereafter, and the public was instructed to quarantine after any travel, even domestically. By the end of March, Nebraska businesses were largely closed to the public, and the state saw its first deaths from COVID-19. All this aside, Nebraska was more liberal in its closing policies than other states. By the end of April, the Nebraska governor and Omaha mayor announced plans to reopen dine-in restaurants, faith communities, and public parks with social distancing and capacity restrictions. Workers in the area were also instructed that they must return to work when called, or they would stop receiving unemployment benefits. These lifted restrictions came in spite of increasing cases and hospitalizations in the community, and continued deaths among vulnerable populations. There were multiple outbreaks in prisons, meatpacking plants, and senior care facilities throughout May and June as restrictions were steadily lifted in the local area. The state also began seeing its first cases of multisystem inflammatory syndrome in children, a severe post-secondary complication in children who previously had COVID-19. Still, the governor insisted on lifting restrictions and, on June 18, 2020, threatened to impose fines and/or rescind federal funding for areas of the state that continued to require masking.

### COVID-19 Related Descriptives

In total, 33 mothers completed all questionnaires of interest between May 19, 2020 and June 29, 2020 (*M* = 80.42 days since approximate lockdown period began, SD = 12.39, range = 64–107 days). Twelve respondents reported that they knew someone who had contracted COVID-19 (*n* = 2 immediate family members; *n* = 1 extended family members; *n* = 4 friends, *n* = 2 coworkers, *n* = 3 acquaintances). Only one respondent reported that they knew someone who had died of COVID-19 (a parent of a close friend); none reported that they had personally contracted COVID-19.

With respect to employment prior to lockdowns, 24 women reported that they worked full-time, 5 were worked part-time, and 3 were unemployed (1 person did not respond). Of the 29 women who were employed, 15 reported a change in work location from working outside the home, to working from home following local lockdowns; 2 respondents dropped from full-time to part-time work, and 1 respondent reported that she lost her job during the lockdown period. Of those employed during the lockdown period, 15% reported that their pay had decreased, 15% were worried about their job security because of the pandemic, and 9% reported that they felt unsafe at work due to the pandemic.

Turning to BAI and BDI total scores, participants had a mean anxiety symptom score of 7.52 (SD = 8.07, range = 0–33) and a mean depressive symptom score of 5.84 (SD = 5.81, range = 0–22). The majority of respondents fell within minimal ranges of both anxiety and depressive symptoms, though 1 respondent registered at moderate clinical depression (scores > 20), and 4 respondents scored in the moderate-to-severe clinical anxiety range (scores > 15).

### Changes in Endorsed Stressors and Impact Scores

**Figure 1:**
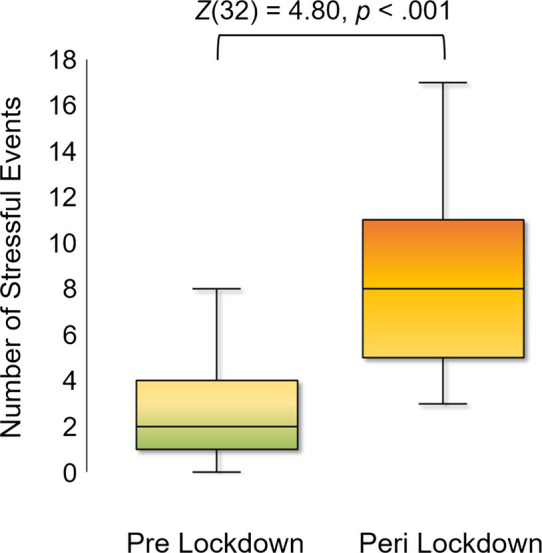
Number of Stressors Endorsed before and after March 15, 2020. Box plots showing the distribution of stressful events endorsed my mothers in the six months prior to local lockdowns beginning (around March 15, 2020; “Pre-lockdown”), and in the time since local lockdowns began (“Post-Lockdown”). A Wilcoxon Z showed a statistically significant increase in the number of stressful events endorsed by mothers since lockdowns began.

Table 1 details the number of respondents who endorsed each stressor on the SRRS before local lockdowns began, and since lockdowns began. On average, mothers reported 2.52 (SD = 2.48, range = 0–10) events in the 6 months prior to lockdowns, versus an average of 8.00 (SD = 3.59, range = 3–17) events since lockdowns began. The increase in the number of endorsed stressors among mothers was significant (*Z*(32) = 4.80, *p* < 0.001; Figure 1). Of note, the three most commonly endorsed stressors before lockdowns began were: (1) mortgage (54.55%), (2) vacation (42.42%), and (3) change in financial state (21.21%). In contrast, the top three most commonly endorsed stressors since lockdowns began were: (1) changes in social activities (90.91%), (2) changes in the number of family get-togethers (87.88%), and (3) changes in recreations (69.70%). Interestingly, there was only one stressor that was endorsed by at least half of the sample prior to lockdowns, whereas there were 6 stressors reported by a majority of respondents since lockdowns began (Table 1).

**Table 1:** Number of participants and percentage of sample who endorsed each stressor from the Social Readjustment Rating Scale (SRRS) before and since local lockdowns began, around March 15, 2020, along with associated impact scores for each stressor.

Impact score	Event	Before 3/15		Since 3/15	
		*N*	%	*N*	%
100	Death of spouse	1	3.03%	0	0%
73	Divorce	1	3.03%	0	0%
65	Marital Separation	0	0%	0	0%
63	Jail Term	0	0%	0	0%
63	Death of close family member	0	0%	2	6.06%
53	Personal injury of illness	0	0%	3	9.09%
50	Marriage	0	0%	0	0%
47	Fired at work	0	0%	0	0%
45	Marital reconciliation	0	0%	0	0%
45	Retirement	0	0%	0	0%
44	Change in health of family member	0	0%	5	15.15%
40	Pregnancy	0	0%	0	0%
39	Sex difficulties	4	12.12%	3	9.09%
39	Gain of a new family member	2	6.06%	2	6.06%
39	Business readjustment	3	9.09%	9	27.27%
38	Change in financial state	7	21.21%	5	15.15%
37	Death of a close friend	2	6.06%	2	6.06%
36	Change to a different line of work	1	3.03%	2	6.06%
35	Change in number of arguments with spouse	1	3.03%	4	12.12%
31	Mortgage over $20,000	18	54.55%	17	51.52%
30	Foreclosure of mortgage or loan	0	0%	0	0%
29	Change in responsibilities at work	3	9.09%	15	45.45%
29	Son or daughter leaving home	4	12.12%	2	6.06%
29	Trouble with in-laws	2	6.06%	3	9.09%
28	Outstanding personal achievement	4	12.12%	1	3.03%
26	Spouse begins or stops work	1	3.03%	0	0%
26	Begin or end school	0	0%	9	27.27%
25	Change in living conditions	1	3.03%	2	6.06%
24	Revisions of personal habits	2	6.06%	13	39.39%
23	Trouble with boss	1	3.03%	1	3.03%
20	Change in work hours or conditions	1	3.03%	19	57.58%
20	Change in residence	2	6.06%	1	3.03%
20	Change in schools	0	0%	2	6.06%
19	Change in recreations	0	0%	23	69.70%
19	Change in church activities	1	3.03%	21	63.64%
19	Change in social activities	1	3.03%	30	90.91%
17	Mortgage or loan less than $20,000	2	6.06%	1	3.03%
16	Change in sleeping habits	2	6.06%	13	39.39%
15	Change in number of family get-togethers	1	3.03%	28	84.85%
15	Change in eating habits	1	3.03%	12	36.36%
13	Vacation	14	42.42%	5	15.15%
11	Minor violation of the law	0	0%	0	0%

**Figure 2:**
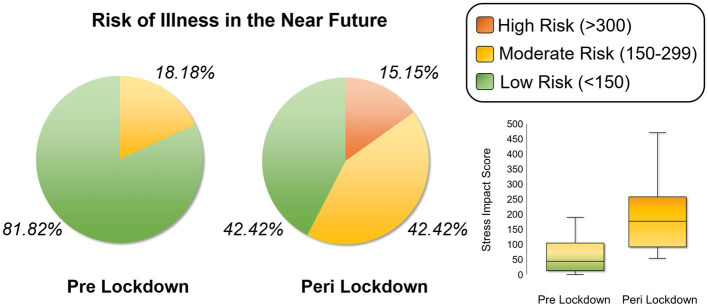
Relative Risk of Stress-Induced Physical Illness in the Near Future. Pie charts showing the proportion of the sample of mothers who were at low risk (30% chance), moderate risk (50% chance) and high risk (80% chance) of contracting a physical illness in the near future based on stress impact scores computed from the Social Readjustment Rating Scale (SRRS) prior to lockdowns versus since lockdowns began. Box plots in the lower right corner demonstrate the distribution of total stress impact scores for the two time periods of interest.

We calculated total impact scores per person based on endorsed stressors pre-and post-lockdown. Women’s average impact scores were 71.64 (SD = 75.51, range = 0–285) pre-lockdown, with 81.82% of mothers falling into the “low risk” category for contracting physical illness in the near future, 18.18% in the “moderate risk” category, and none in the “high risk” category. Average stress impact scores markedly increased to 189.61 (SD = 101.24, range = 60–476) since lockdowns began. Only 42.42% of mothers were still “low risk” for future illness, with another 42.42% at “moderate risk,” and 15.15% at “high risk” of contracting a stress-related physical illness in the near future (Figure 2).

**Figure 3:**
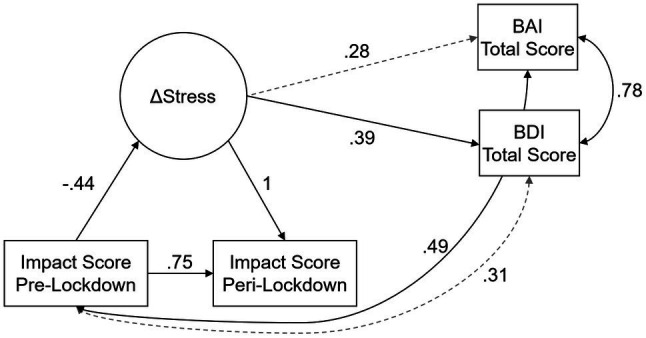
Latent Change Score Model of Stress, Anxiety, and Depression. The latent change score model of changes in stress impact (ΔStress) predicting self-reported anxiety Beck Anxiety Inventory (BAI) and depression Beck Depression Inventory (BDI) scores. Double-headed arrows represent correlations, and single-headed arrows represent unidirectional effects; solid black lines indicate statistically significant effects at the *p* < 0.05 level, and dashed gray lines indicate non-statistically significant effects. Coefficients reported in the figure are standardized effects.

**Figure 4:**
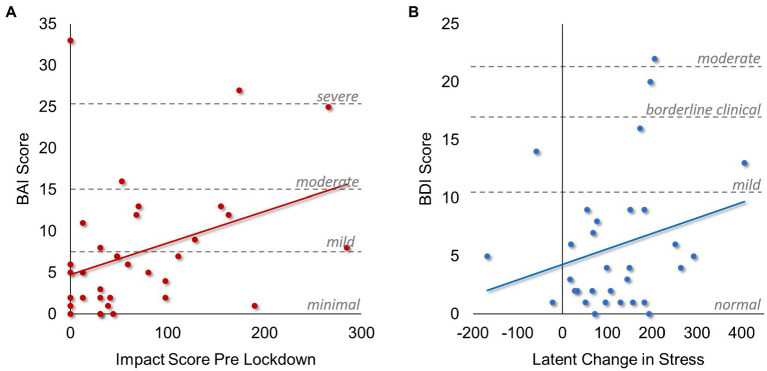
Associations between Stress, Anxiety, and Depression. Scatterplots demonstrating significant associations found in the latent change score model of life stress impact, depression, and anxiety. **(A)** Associations between self-reported anxiety scores (BAI) and life stress impact scores prior to local lockdowns beginning. Dashed gray lines and associated labels demonstrate different clinical cutoff criteria for anxiety scores obtained from the BAI, with “moderate” and “severe” categories considered clinically significant. **(B)** Associations between self-reported depression scores (BDI) and the latent change in life stress impact scores assessed as the difference between post-and pre-lockdown stress impact scores. Dashed gray lines and associated labels demonstrate different clinical cutoff criteria for depression scores obtained from the BDI, with the “moderate” category considered clinically significant.

We fit a latent change score model to assess differences in impact scores (Figure 3). The latent change variable of stress impact scores had a mean of 163.58 (*p* < 0.001), though there was significant individual variability in differences between pre-and post-lockdown impact scores (*σ*^2^ = 5529.88, *p* < 0.001). Interestingly, the latent change in impact scores was related to pre-lockdown impacts, such that individuals who had higher impact scores pre-lockdowns tended to see less change in impact scores post-lockdowns (*β* = −0.44, *b* = −0.64, *p* < 0.005). These conclusions held when adding in relationships to BAI and BDI total scores. Additionally, we found that greater latent change in stress impact was associated with significantly higher BDI scores during the pandemic (*β* = 0.39, b = 0.020, *p* = 0.036; Figure 4A). There was no significant correlation between BDI scores and pre-lockdown stress impact scores. In contrast, BAI scores were not significantly related to the latent change in stress impact, but they were related to pre-lockdown impact scores (*r* = 0.49, *p* = 0.001; Figure 4B). These data suggest that mothers who had greater pre-lockdown stress impact scores tended to experience greater anxiety symptoms during the lockdown period. Finally, BAI and BDI scores were significantly correlated (*r* = 0.78, *p* < 0.001), indicating that individuals who reported higher anxiety symptoms also tended to report higher depressive symptoms.

## Discussion

In the present study, we examined the extent to which the number and impact of life stressors changed among mothers prior to-versus since COVID-19 pandemic-related lockdowns began in a small sample of mothers. One of our key findings was that mothers reported significantly more stressful events in the 2-to 3-month period since lockdowns began, relative to the 6-months prior to local lockdowns. Moreover, there was a marked increase in the proportion of women at a moderate-to-high risk of contracting a physical illness in the near future as a result of their stress. Importantly, we found that the *degree of change* in stress impact was associated with higher self-reported post-lockdown depressive symptom scores, whereas post-lockdown anxiety symptoms were associated more specifically with *pre-pandemic* stress impact.

First and foremost, we found significant increases in stress impact scores among mothers. Prior to the pandemic, mothers endorsed relatively few stressful events, and 82% of the sample was at low risk for developing a physical illness in the near future as a result of their stress. This was in stark contrast to stress-related risks during the pandemic period, with almost 58% of mothers at moderate-to-high risk of illness. Researchers have long known the associations between stress and risk for disease with respect to both chronic and acute stressors (Holmes and Masuda, 1973; Christie-Seely, 1983; Cohen et al., 2007, 2019). Some of the physical health burden associated with stress is a direct result of changes in personal health habits like experiencing frequent sleep disturbances, engaging in maladaptive coping mechanisms like smoking, and adopting poor eating habits (Sobel and Markov, 2005; Cohen et al., 2007, 2019). Over one-third of mothers in the present study indicated changes in sleep, eating, and personal habits since the pandemic began. Fortunately, many of these habits can be modified to promote physical and mental health. Notably, these drastic increases in stress prevailed despite the relatively high job security reported by mothers in the present study. It is likely that mothers in more economically precarious situations would report even greater stress. Further work should be done examining a more socio-economically heterogeneous sample of mothers.

Critically, we found that the *degree of change* in life stress was associated with mothers’ post-lockdown depressive symptoms, whereas *pre-pandemic* stress was associated with post-lockdown anxiety symptoms. This is an important distinction that uniquely contributes to the extant literature. Several studies have reported that the pandemic has exacerbated symptoms in those with pre-existing conditions, and has fostered the onset of psychological symptoms in those without prior histories (Asmundson et al., 2020; Ho et al., 2020; Mazza et al., 2020; Ornell et al., 2020), though the discussion has been broadly applied to both anxious and depressive symptoms. Our data specifically suggest that depressive symptoms may be a more novel psychological phenomenon among mothers during the pandemic, whereas anxiety symptoms may be more linked to prior cumulative life stress. Such nuance could help inform practitioners to better target specific aspects of life stress that may have more chronically contributed to anxiety symptoms, versus those that more acutely affected depressive symptoms among mothers. Interestingly, recent work reported that working mothers generally had greater anxiety than working women without children, though this effect was ameliorated by self-reported resilience (Benassi et al., 2020). Thus, it is possible that other individual differences in stress management and perceptions of stress may have played an important role in our current findings. Studies in larger samples should further investigate individual differences that contribute to unique experiences and stress responses during the pandemic.

Finally, our findings largely corroborate prior literature reporting increased stress among mothers during the COVID-19 pandemic (Fisher et al., 2020; Patrick et al., 2020; Spinelli et al., 2020). Mothers consistently endorsed a greater number of stressors since local lockdowns began, which was coupled with significantly greater life stress impact. This finding is unsurprising when considering the most commonly-reported events during the lockdown period; changes in social and family gatherings, revisions to personal routines, changes in recreations and church habits, and shifts in work were all commonly endorsed in the present study. Essentially, as COVID-19 lockdowns began, people around the globe were required to socially distance themselves from those outside of their immediate family, to work remotely or stop working, and to adopt new hygiene habits, including extra hand washing and sanitizing, and donning a mask in public (Taylor, 2019; Fisher et al., 2020; Xiong et al., 2020). All these changes in lifestyle cumulatively contribute to one’s overall stress and align with the commonly-reported events. Other studies specifically examining mothers have found similar reductions in mental wellness during the pandemic (Di Giorgio et al., 2020; Shevlin et al., 2020).

This study was not without limitations. Critically, this was a small-*N* study meant to shed light on the nuanced impacts of the pandemic on mothers’ mental health. With a small sample sizes come challenges with generalizability to the broader population, as well as the risk that we may have missed important effects due to the underpowered sample. Our data provide *preliminary* evidence that stressors introduced by local lockdowns versus these that were already present prior to lockdowns were differentially associated with mothers’ anxious versus depressive symptoms. However, further work on a larger scale is critical to identify whether these trends are consistent in socioeconomically, racially, and ethnically diverse mothers across a broad geographic distribution. Secondly, this study was conducted retrospectively. There is the possibility of recall bias in responses. Ideally, researchers who were already conducting large-scale studies of mental health will be able to examine “true” changes in stressors based on data collected prior to-versus following local lockdowns. It would also be fruitful to explore responses on self-reported questionnaires against those collected interview-style from trained clinicians. Finally, we only assessed broad stressors, rather than querying stressors specific to the pandemic. It is possible (and likely) that the surveys utilized herein did not capture the full extent of stressors introduced by the pandemic. However, there are numerous works already published which specifically probe the pandemic. The current study unique assessed changes in daily life stressors rather than pandemic-specific disruptions, which may be differentially associated with anxious and depressive symptomatologies.

### Implications for Policy

These preliminary data provide evidence that (1) mothers’ stress during the pandemic increased to alarming levels, putting them at risk of contracting physical illness, and (2) increases in stressors during local lockdowns are significantly associated with mothers’ depressive symptoms. Although further work is needed in larger samples, these data suggest that the pandemic may have severely impacted mothers’ health and well-being. Policymakers should consider the impact of the current, as well as future pandemics, on mental and physical wellness among mothers. There is a critical need to develop additional programs to monitor and support mothers’ physical wellness, as well as to provide safe assistance for challenges contributing to mental health like inadequate childcare, difficulties with work-home balances, and social isolation. It would be beneficial to conduct focus groups among mothers from diverse backgrounds and with diverse needs to better understand what programs and tools would be most beneficial in order to effectively direct resources to the most impactful areas. Keeping mothers healthy should be a public health priority. That said, increasing accessibility to telehealth appointments, creating online social/support groups for mothers, and offering better solutions for distance learning to reduce the burden on parents are all potential solutions to promote mental wellness. Additionally, better government resources for childcare, and more protections for workers who are also parents is a necessity so that parents are better able to balance work and family, especially when alternate forms of childcare are not tenable.

### Conclusion

The present study highlights the need for mental health resources to address psychological distress among mothers during the COVID-19 pandemic. Herein, we reported significant increases in life stress which were uniquely associated with depression symptoms, extending into clinical severity for some. Scientists have described a “pandemic of fear and stress” concomitant with the COVID-19 pandemic; people are isolated from their typical social support networks, and public health expenditures are focused on fighting the pathogen rather than managing psychological distress (Ornell et al., 2020). All of this is happening in a time when an estimated 12 million Americans had unmet mental health needs prior to the pandemic, before many lost their employer-provided health insurance (Marques et al., 2020). As pandemics become more frequent in an increasingly globalized world, we as a society must better understand the mental health repercussions of the challenges ahead. The present study highlights the need for mothers to find safe ways to connect with social support networks and/or cope with the more acute effects of life stress resulting from the pandemic.

## Data Availability Statement

The raw data supporting the conclusions of this article will be made available by the authors, without undue reservation.

## Ethics Statement

The studies involving human participants were reviewed and approved by University of Nebraska Medical Center Institutional Review Board. The patients/participants provided their written informed consent to participate in this study.

## Author Contributions

BT, SW, AB-B, and TW contributed to the conception and design of the study. MF, HJ, and MW organized the database, recruited participants, and acquired the data. BT performed the statistical analysis and wrote the original manuscript. All authors contributed to the manuscript revision, read, and approved the submitted version.

### Conflict of Interest

The authors declare that the research was conducted in the absence of any commercial or financial relationships that could be construed as a potential conflict of interest.

## Footnotes

^1^coronavirus.jhu.edu

^2^https://omaha.com/news/state-and-regional/covid-19-timeline-of-the-pandemic-in-nebraska/article_f3940100-2bf5-5327-a129-0ec45e037984.html
